# Temperature mapping of operating nanoscale devices by scanning probe thermometry

**DOI:** 10.1038/ncomms10874

**Published:** 2016-03-03

**Authors:** Fabian Menges, Philipp Mensch, Heinz Schmid, Heike Riel, Andreas Stemmer, Bernd Gotsmann

**Affiliations:** 1 IBM Research—Zurich, Säumerstrasse 4, CH-8803 Rüschlikon, Switzerland; 2 Nanotechnology Group, ETH Zürich, Säumerstrasse 4, CH-8803 Rüschlikon, Switzerland

## Abstract

Imaging temperature fields at the nanoscale is a central challenge in various areas of science and technology. Nanoscopic hotspots, such as those observed in integrated circuits or plasmonic nanostructures, can be used to modify the local properties of matter, govern physical processes, activate chemical reactions and trigger biological mechanisms in living organisms. The development of high-resolution thermometry techniques is essential for understanding local thermal non-equilibrium processes during the operation of numerous nanoscale devices. Here we present a technique to map temperature fields using a scanning thermal microscope. Our method permits the elimination of tip–sample contact-related artefacts, a major hurdle that so far has limited the use of scanning probe microscopy for nanoscale thermometry. We map local Peltier effects at the metal–semiconductor contacts to an indium arsenide nanowire and self-heating of a metal interconnect with 7 mK and sub-10 nm spatial temperature resolution.

## Abstract (web summary)

Thermometry using scanning probe techniques allows for the thermal imaging and characterization of devices with nanoscale resolution, however can be hindered by contact-related artefacts. Here, the authors demonstrate a thermal scanning probe approach which eliminates contact-resistance effects.

## Graphical abstract

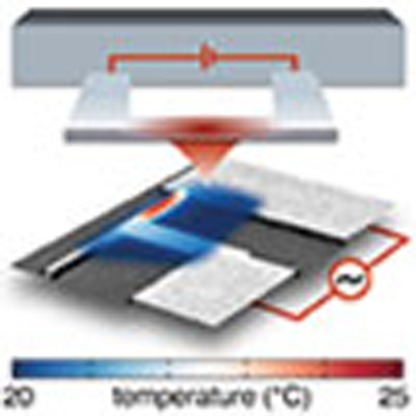

A key challenge in nanoscience is the development of versatile techniques to map variations in temperature with nanometre-scale spatial resolution^1^,^2^,^3^,^4^,^5^,^6^,^7^. Various studies have addressed this using techniques such as Raman^1^, fluorescence^8^,^9^,^10^ and luminescence thermometry^3^. These methods, however, suffer from an optical diffraction-limited spatial resolution that is not sufficient to characterize hotspots only a few nanometres in size^11^. A recent transmission electron microscopy approach overcomes this optical diffraction limit^12^, but is only applicable to thin samples (<100 nm) of certain materials (specific metals and semiconductors). To address the lack of versatile, high-resolution thermometry techniques, scanning probe-based methods have been explored^13^,^14^,^15^,^16^,^17^,^18^,^19^,^20^,^21^,^22^,^23^,^24^,^25^,^26^,^27^,^28^. Thermal scanning probe measurements, however, typically suffer from contact-related artefacts^13^,^14^,^29^,^30^ that restrict their applicability for nanoscale thermometry.

Unlike macroscopic contact thermometers, they typically not infer temperature by measuring only one physical property, for example, the electrical resistance, of a calibrated sensor in equilibrium with the system of interest. Instead, they detect a heat-flux-related signal across a tip–sample contact 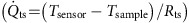 that is not only proportional to the temperature difference between a probe sensor (*T*_sensor_) and a sample (*T*_sample_), but also influenced by an unknown thermal contact resistance (*R*_ts_(*T*))^29^,^30^. The well-established concept of equilibrium contact thermometry cannot be easily adapted to the nanoscale, as it would require *R*_ts_ to be small compared with the resistance between the sensor and its thermal reservoir (*R*_cl_), a condition that practically cannot be achieved for high-resolution scanning probes forming nanoscopic contacts^18^,^19^,^22^,^23^,^24^,^25^.

To measure the temperature in such a non-equilibrium situation, one has to account for the thermal coupling strength (given by the value of *R*_ts_) between sample and sensor. This demand is comparable to that for an infrared thermometer, requiring emissivity corrections to quantify the temperature of a non-ideal black body as potential that governs a (radiative) heat flux. Previous attempts addressing this issue on the nanoscale including a two-pass method^23^ and dynamic adjustment of the probe sensor temperature^24^,^31^ are complicated to implement and could not demonstrate reliable temperature mapping in those cases where the surface topography is inhomogeneous. This is mainly because *R*_ts_ of a nanoscopic contact is not only difficult to predict^13^,^14^,^29^,^30^ but a position-dependent property that varies dynamically during the imaging of a surface.

Here we first elucidate the challenge of using thermal scanning probes for nanoscale thermometry, before we present an approach that enables the elimination of contact-related artefacts. We illustrate how the perturbing variations of *R*_ts_ can be separated from sample temperature variations by simultaneously probing a time-dependent and a time-averaged heat flux signal between a self-heated scanning probe sensor and a temperature-modulated sample.

## Results

### Self-heating of a metal interconnect

**Figure 1:**
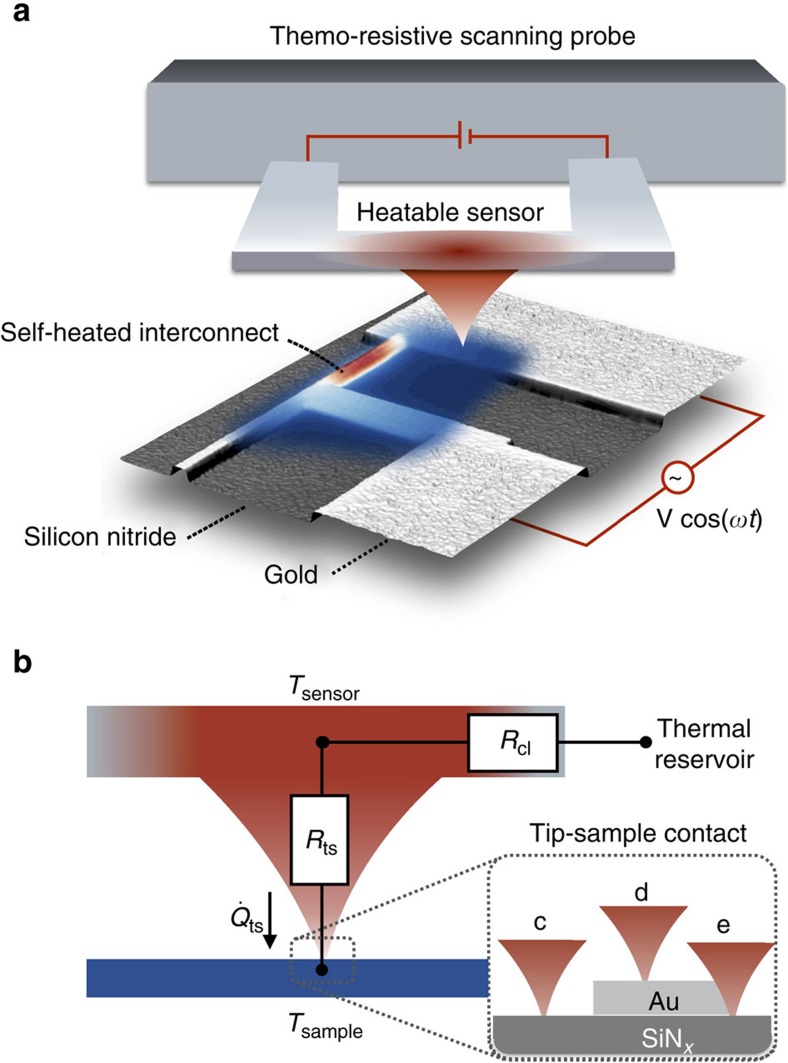
Illustration of the experiment. (**a**) Schematic of the thermoresistive scanning probe in contact with a self-heated gold (Au) interconnect (∼100 nm wide) on a silicon (111) substrate wafer covered with 150 nm of amorphous silicon nitride (SiN_*x*_). An alternating voltage bias (*V* cos(*ωt*)) is applied to modulate the sample temperature. The sample temperature field (red–blue colour scheme indicates the temperature from hot to cold) is inferred by simultaneously probing a time-dependent and a time-averaged heat flux signal between the heatable (red coloured) sensor and the temperature-modulated sample. (**b**) The sensor is decoupled from its thermal reservoir by the thermal resistance of the cantilever (*R*_cl_). The tip–sample heat flux 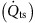 is not only a function of the temperature difference between the sensor (*T*_sensor_) and sample (*T*_sample_), but also of the tip–sample contact thermal resistance (*R*_ts_). Changes of the tip–sample contact due to (c–d) the material in contact with the tip, (d–e) the tip–sample contact area and tribological effects cause variations of *R*_ts_.

**Figure 2:**
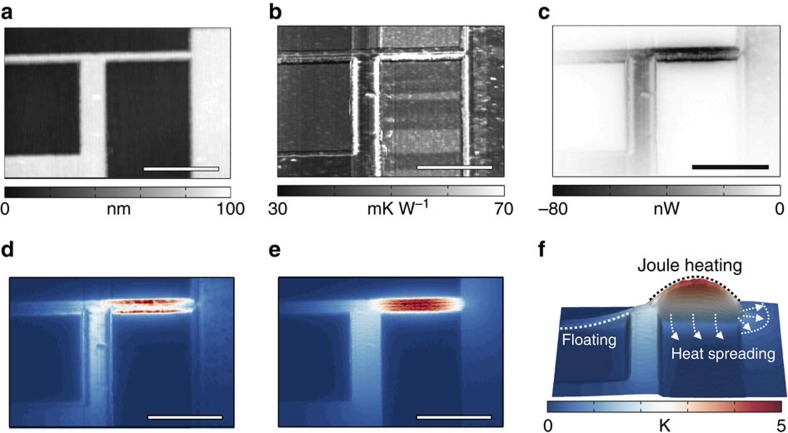
Self-heating of a nanoscale metal interconnect structure. A bipolar voltage bias is applied to the electrical interconnect shown in (**a**) as height image (scale bar, 1 μm). Joule heating of the structure is characterized by simultaneously mapping local variations in (**b**) the tip–sample thermal resistance (*R*_ts_) and (**c**) the tip–sample heat flux (*Q*_ts,AC_). Previous approaches inferred local temperature fields using a constant, position-independent (*x*,*y*) tip–sample thermal resistance (for example, the mean of *R*_ts_(*x*, *y*)). This leads to significant artefacts, illustrated as (**d**) an apparent temperature distribution. By combining position-dependent thermal resistance variations with the local heat flux maps, we can calculate (**e**) the true sample temperature rise (Δ*T*_sample_). This relative temperature increase can be encoded in terms of both height and colour as shown (**f**), to illustrate the characteristic (parabolic) Joule heating along the interconnect segment, as well as heat spreading (indicated by the white dotted arrows and lines) into the SiN_*x*_/Si substrate and the electrically floating interconnect segment. It is noteworthy that the colourbar applies also to **d**,**e**.

To demonstrate the technique, we first characterize the Joule heating of a gold interconnect structure shown schematically in Fig. 1 and as an atomic force micrograph in Fig. 2a. An alternating voltage bias (±3 V) is applied to the interconnect, inducing an AC current of ±2.4 mA (see Supplementary Fig. 1 for a detailed schematic of the data acquisition). When using sufficiently small frequency (*f*=*ω*/(2*π*)=10 kHz), the applied voltage bias leads to a steady-state temperature increase and a continuous modulation of the interconnect temperature (*T*_sample_=*T*_RT_+Δ*T*_sample_(1+sin(2*ωt*))) at twice the excitation frequency, with *T*_RT_ denoting room temperature. The change in sample temperature (Δ*T*_sample_) affects the tip–sample heat flux 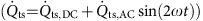, which is related to the measured electrical power (*P*_el_) dissipated in the probe sensor and also by the sensor temperature (*T*_sensor_=*T*_sensor,DC_+*T*_sensor,AC_sin(2*ωt*)), which is proportional to the electrical resistance (*R*) of the sensor (see the Supplementary Note 1 for the derivation of the relation between power, resistance and temperature of the probe). By simultaneously measuring the temperature-dependent steady-state (DC) and the alternating (AC) response of the probe sensor, the sample temperature increase can be derived as


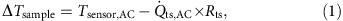


with


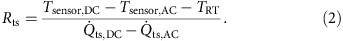


Importantly, a part of the DC sensor temperature component (*T*_sensor,DC_) is set by the sensing voltage bias applied to the scanning probe, which typically heats the sensor to about 20–200 K above *T*_RT_. As a result, *T*_sensor,DC_ and *T*_sensor,AC_ are not the same, and the two unknown Δ*T*_sample_ and *R*_ts_ can be extracted simultaneously from the two measurable quantities *T*_sensor,DC_ and *T*_sensor,AC_ via equations (1) and (2) for each pixel of an image under the assumption that, for a given scan position, *R*_ts_ is approximately constant within the range of sample temperature modulation. Under this assumption, *R*_ts_ can be directly expressed as a ratio between the measured heat fluxes and sensor temperatures, while previously *R*_ts_ had to be either estimated by modelling simulations^14^,^15^,^18^,^19^,^22^,^25^ or be determined from reference measurements^23^. The fact that we can avoid this step reduces systematic and propagated measurement errors dramatically.

Figure 2b shows the thermal resistance (*R*_ts_) map recorded simultaneously to the AC tip–sample heat flux 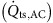, while raster scanning the probe in contact with the surface. Measurements were conducted in a high-vacuum environment (<10^−6^ mbar) that diminishes parasitic heat conduction between the probe sensor and the sample via air-mediated conduction/convection. Large variations in *R*_ts_ can be observed in correlation with topography features that change the tip–sample contact area. These features perturb 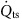, as evident from the signal variations at the interconnect edges in Fig. 2c.

Previous approaches for inferring temperature maps using scanning thermal microscopy (SThM) did not fully account for these contact-related effects, but typically assumed a position-independent value for *R*_ts_ (refs 14, 15, 18, 19, 22, 25) to transform a heat-flux-related signal to a temperature field map. Such a procedure, however, can lead to significant systematic errors as shown in the ‘apparent' temperature map in Fig. 2d. The section of the interconnect that is heated most even shows a reduced temperature in the centre of the metal line with respect to its edges, which is highly implausible. By accounting for position-dependent variations of *R*_ts_, however, we can obtain a corrected temperature map (see Fig. 2e). The rather homogenous temperature field and the absence of nanoscopic, contact-related features in Fig. 2e, are important results of our work and highlighting the benefits of the scanning probe thermometry approach presented here. As this technique is applicable to various kind of material surfaces, it also enables to map the temperature field extending around the heated interconnect. To illustrate this, Fig. 2f shows the temperature distribution of Fig. 2e encoded in terms of both height and colour. Not only the self-heating of the thinnest interconnect segment exhibiting the typical, nearly parabolic temperature profile due to Joule heating but also the temperature decay into the floating interconnect segment and the spreading of heat into the substrate are resolved. This real-space temperature field information can be particularly valuable when comparing with models and simulations^18^,^23^, and potentially provides further insight into the thermal coupling between the interconnect and the substrate.

### Self-heating of an InAs nanowire

**Figure 3:**
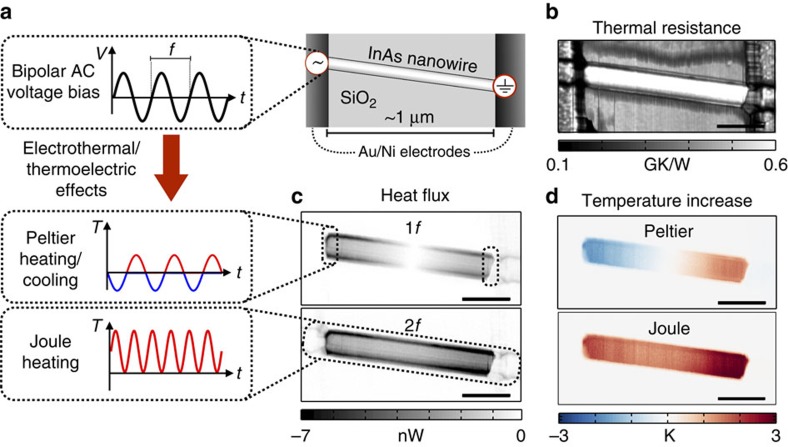
Direct imaging of local Joule and Peltier effects of a self-heated nanowire. (**a**) A bipolar AC voltage bias (0.7 V, frequency *f*=*ω*/(2*π*)=10 kHz) is applied to the InAs nanowire (∼120 nm wide) contacted by metal (Au/Ni) electrodes, leading to Peltier heating/cooling at the metal–semiconductor contacts and Joule heating in the nanowire. (**b**) Tip–sample thermal resistance (*R*_ts_) image (scale bar, 300 nm) recorded simultaneously to (**c**) the tip–sample heat flux 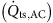 at two different frequencies (1*f* and 2*f*) corresponding to Peltier and Joule effects, respectively. (**d**) Instantaneous Peltier and Joule distributions of temperature at maximum modulation amplitudes, calculated by combining the thermal resistance and the heat flux maps, which enables us to eliminate tip–sample contact-related artefact in regions where the surface topography is inhomogeneous and the material composition varies. It is noteworthy that there is no net Peltier effect along the nanowire in time average.

Having established a reliable approach to image nanoscopic temperature fields, we use it to study self-heating of an indium arsenide (InAs) nanowire^32^. Again, a bipolar AC voltage bias is applied (see Fig. 3a), inducing not only Joule heating in the nanowire, but also Peltier heating/cooling at the metal–semiconductor interfaces. In contrast to previous SThM approaches mapping only overall temperature fields, our method can be applied to study both effects separately, an important option that facilitates direct characterization of thermoelectric effects in real space that might find relevance for the characterization of operating logic, memory- and energy-harvesting devices. For a linear device (for example, an ohmic resistor), the two effects can be distinguished in a single measurement scan (see ref. 33 and ref. 26), because the Peltier effect has a linear dependence 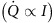 on an electrical current, matching the frequency of the applied voltage bias (see Fig. 3a), whereas Joule heating has a quadratic response 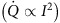 that can be detected in the higher harmonic response (2*f*) of the probe sensor. Applying the same procedure as described for the metal interconnect, we can simultaneously map variations in the thermal resistance (see Fig. 3b) and variations in the tip–sample heat flux (see Fig. 3c), now separated for the two frequencies (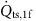 and 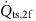) corresponding to Peltier and Joule effects, respectively. The assignment of the (1*f* and 2*f*) signals to the heat generation due to Peltier and Joule effects, respectively, is supported by an analysis of the observed effects as a function of voltage bias amplitude (see Supplementary Note 2 and Supplementary Fig. 3). By combining the thermal resistance and heat flux measurements, we can successfully eliminate tip–sample contact-related artefacts and calculate the sample's temperature field according to equations (1) and (2) expanded for two frequencies.

The instantaneous Peltier temperature field, shown in Fig. 3d, induced by net heat generation or extraction at the nanowire–metal contacts exhibits a symmetrical, almost linear temperature profile along the nanowire length with a vanishing temperature change in the centre (see Supplementary Note 3 and Supplementary Fig. 4 for the related phase signals). It is noteworthy that the Peltier temperature at a certain phase is shown; in time average, there is no heating or cooling of the nanowire in response to the bipolar AC excitation. Conversely, the Joule-heating-related temperature increase of the nanowire (see Fig. 3d) does not show a nearly parabolic temperature distribution as observed for the self-heated metal interconnect, but indicates a temperature increase towards the electrical contacts (see also Supplementary Fig. 2). This could stem from either a very non-homogenous thermal coupling of the respective nanowire sections to the substrate or enhanced Joule heating near the Au/Ni-InAs contacts. As there is no indication of a non-uniform thermal coupling to the substrate in the Peltier measurement, we conclude that the Joule heating along this particular nanowire must be unevenly distributed, favouring the nanowire–metal contact regions.

## Discussion

To discuss the method presented here, we first consider the sample-temperature resolution of our measurements. As argued above, the sample-temperature resolution is directly correlated to the sensitivity for variations in heat flux. We characterized the voltage noise-limited temperature resolution (*δT*_res−probe_) of our thermal probes, which range from 1 to 100 μK 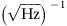 as a function of sensor temperature and operation frequency. For the measurements presented, we calculate a temperature resolution of *δT*_res−probe_=2 μK 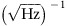 and 20 μK 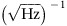 for the interconnect and nanowire experiments, respectively, which is two to three orders of magnitude larger than in a recent nanoscale thermometry approach of similar spatial resolution based on plasmon energy expansion thermometry^12^. With a probe sensor thermal resistance *R*_cl_ on the order of 2 × 10^5^ K W^−1^, this translates into a sub-100 pW heat flux resolution 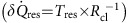 at 20 kHz operation frequency. A comparable heat flux resolution has previously only been achieved by MEMS nanocalorimeters^34^, which, however, do not provide local imaging functionality. Achieving this high sensitivity is important, as it allows the total heat flux to be limited to values that are small enough not to perturb the heat generation in the sample volume beneath the tip (<100 nW for the nanowire experiment). The heat flux used in our experiments is comparable to that used in typical non-contact, radiative heat flux experiments based on Raman or fluorescence thermometry, thus ruling out one of the main concerns against using contact-based methods for nanoscale thermometry^3^,^12^.

The sample temperature resolution is limited by the heat flux resolution of the thermal scanning probe system and could be estimated based on the intrinsic resolution of the probe sensor and the tip–sample thermal resistance 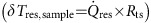. Typically, either the intrinsic noise in the sensor circuitry^22^ or the noise extracted from nominally flat regions in the image data^23^,^28^ are used to quantify the resolution. The latter is a more conservative approach, as it includes additional noise sources affecting the measured sample temperature field, such as mechanical noise of the tip–sample contact or changes at the tip apex. Therefore, we prefer to directly extract the sample temperature resolution as root mean square noises from the experimental data of Figs 2e and 3f, reaching 7 and 85 mK, respectively. These sample temperature resolutions have to be considered in the context of lateral resolution, which is directly linked to the value of *R*_ts_ via the size of the tip–sample contact. The sample temperature resolution of the nanowire experiments is less than those of the metal interconnect studies, whereas spatial resolution is higher. In the nanowire experiment, we observe a tip–sample thermal resistance (up to *R*_ts_=6 × 10^8^ K W^−1^) similar to that of a previous SThM resolution demonstration of 6 nm^35^, which also matches the smallest features observable in the nanowire scans. Therefore, we conclude that the method is suitable for achieving a sub-10 mK temperature resolution at sub-10 nm lateral temperature resolution with the microscope used.

It is noteworthy that a meaningful demonstration of a spatial temperature resolution in SThM measurements is challenging. Ideally, it requires a step-like temperature variation in the absence of any other variation influence the heat flux-related measurement signal such as a material- or topography-related change in the tip–sample thermal resistance. However, using contact model estimations to calculate the size of the mechanical contact between the tip and sample are oftentimes inherently inaccurate and can be in contradiction to the heat flux resolution, which is a strong function of contact size. Therefore, a meaningful resolution definition for SThM measurements should be based on the tip–sample heat flux resolution, as heat flux-related signals are measured and directly reflecting the tradeoff between sample temperature resolution and spatial resolution via their common dependence on the tip–sample contact size. In our experiments, we can extract a heat flux resolution of 140 pW in a 100-Hz bandwidth, which is a factor of 25 or more improvement compared with previous SThM measurements^22^,^23^,^28^.

Finally, we may also discuss limitations of the scanning probe thermometry approach presented. Although the technique described can be easily implemented, it is limited to cases where the sample temperature can be modulated, for example, by electrical or optical means. Steady heat sources may not be fully characterized by the presented approach and alternative techniques, for example, based on multiple measurements^23^,^24^ might be applied to those cases. Although the presented modulation-based thermometry approach simplifies the temperature field characterization as all signals can be characterized in a single measurement scan, it comes on the additional experimental effort to create a temperature modulation of known frequency in the first step. As the modulation frequency can be flexible chosen, even the transient regime of local heat sources might be characterized to extract further information, for example, about the thermal diffusivity of the system, beyond the direct imaging of a steady-state temperature field itself (see Supplementary Discussion and Supplementary Fig. 5). Furthermore, care is needed not to perturb the sample heat source by the measurement heat flux between the scanning probe sensor and the sample. It is noteworthy that this is a common challenge to any thermometry method, even for non-contact-based approaches where optical or electron beam-based sensing fluxes are used for detection.

To summarize, we have introduced a versatile scanning probe thermometry technique to enable quantitative, spatially resolved temperature measurements down to few-nanometre spatial resolution and sub-10 mK temperature resolution. The presented approach accounts for contact-related artefacts, so far limiting the reliability of scanning probe microscopy for nanoscale thermometry, and may promote further understanding of local thermal processes in various areas of nanoscience. It can be used to characterize surfaces of almost arbitrary materials and is expected to have a direct impact on understanding of nanoscale devices dealing with local thermophysical effects such as the spin-Peltier effect^5^, the magnetocaloric effect or the thermoplasmonic effect^9^.

## Methods

### Experimental setup

Experiments where conducted using a custom-built high-vacuum SThM^35^ situated in an electromagnetically shielded, temperature-stabilized laboratory^36^. Our typical cantilevers have a nominal spring constant of 0.15–0.20 N m^−1^ and a resonance frequency in the range of 50 kHz, making them well suited for contact-mode operation. The cantilever is highly phosphorus doped (10^20^ cm^−3^) for good electrical conductivity. The heater region of (4 × 6) μm below the tip has a nominal dopant density of 5 × 10^17^ cm^−3^. The thermal resistance of the cantilever when situated in vacuum is typically 2 × 10^5^ K W^−1^. The thermal resistance is mainly defined by the heat flux from the heater region along the anchor beams of the cantilever into the chip body. The thermal isolation of the heater region is maximized in a tradeoff between the mechanical and thermal properties of the lever. For scanning probe operation, we require a critical thickness of the cantilever, which is already a thin beam of only 300 nm in the region of the hinge. More than 90% of the total power dissipation in the cantilever occurs in the small region of the heater element below the tip. The heater equilibrates in a few microseconds. The electrical resistance is on the order of 1.2 kΩ at room temperature and increases to about 3.3 kΩ at 550 °C. Overall, the heater can be heated up to 1,000 °C. Further details on the cantilever, its design and fabrication are reported by Drechsler *et al*.^37^.

### Sample fabrication

The metal interconnect test structure consists of nanoscale gold interconnects on a silicon (111) substrate waver coated with 150 nm of amorphous silicon nitride (SiN_*x*_). The narrow gold lines (∼100 nm wide) were fabricated via electron-beam lithography (Vistec EBPG5200) and patterned by an etching process.

InAs nanowires were grown by metal-organic vapour-phase epitaxy on a silicon(111) wafer^38^. The nanowire (∼120 nm diameter) was separated from the growth substrate and transferred to a silicon substrate covered with a ∼100-nm-thick silicon oxide. Electrical contacts (Au/Ni) were fabricated by e-beam lithography.

## Additional information

**How to cite this article:** Menges, F. *et al*. Temperature mapping of operating nanoscale devices by scanning probe thermometry. *Nat. Commun.* 7:10874 doi: 10.1038/ncomms10874 (2016).

## Supplementary Material

Supplementary Information Supplementary Figures 1-5, Supplementary Notes 1-3, Supplementary Discussion and Supplementary References
