# Supplementary material for: Temperature mapping of operating nanoscale devices by scanning probe thermometry
Source: Nat Commun. 2016 Mar 3;7:10874. doi: 10.1038/ncomms10874 (PMC4782057; doi:10.1038/ncomms10874)
Supplement: Supplementary Information — Supplementary Figures 1-5, Supplementary Notes 1-3, Supplementary Discussion and Supplementary References [file ncomms10874-s1.pdf]

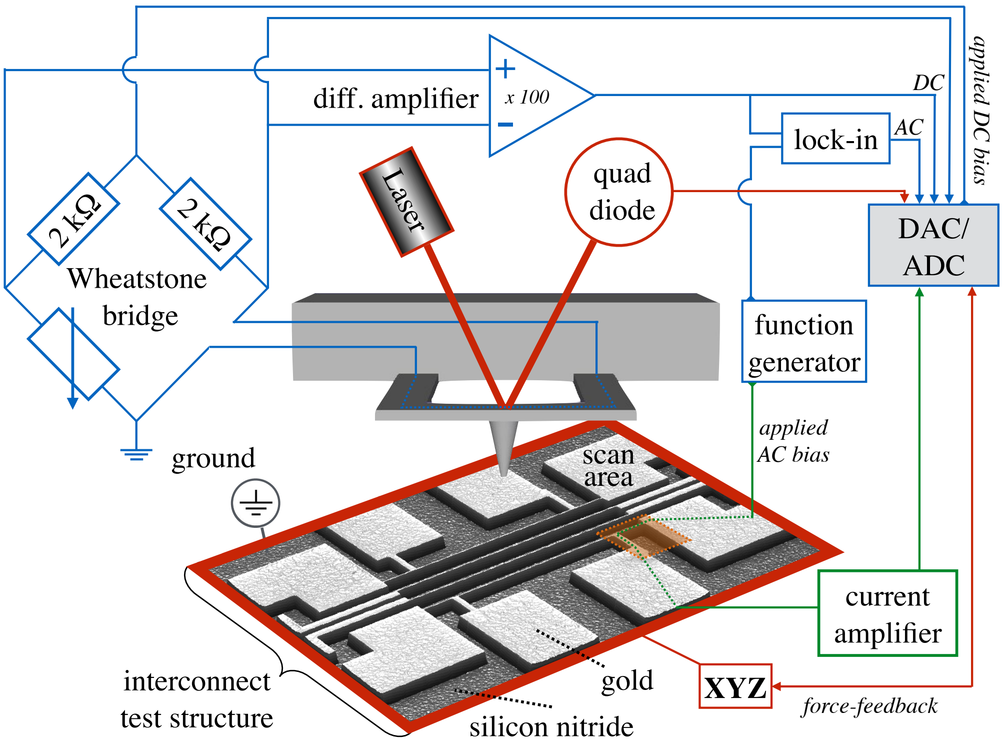


**Supplementary Figure** 1. **Overview of the data acquisition**

(blue) Thermal signals, (red) position signals, and (green) device-related signals. An alternating voltage bias is applied to the gold interconnect segment indicated as scan area via an function generator. The thermal signal of the scanning probe in contact with the sample is acquired by the electrical circuitry indicated in blue, which comprises a Wheatstone bridge, a differential amplifier, and a lock-in amplifier. All signals are applied via the digital-analog output (DAC) and acquired via the analog-digital input (ADC) of an ADwin Pro real-time processor.

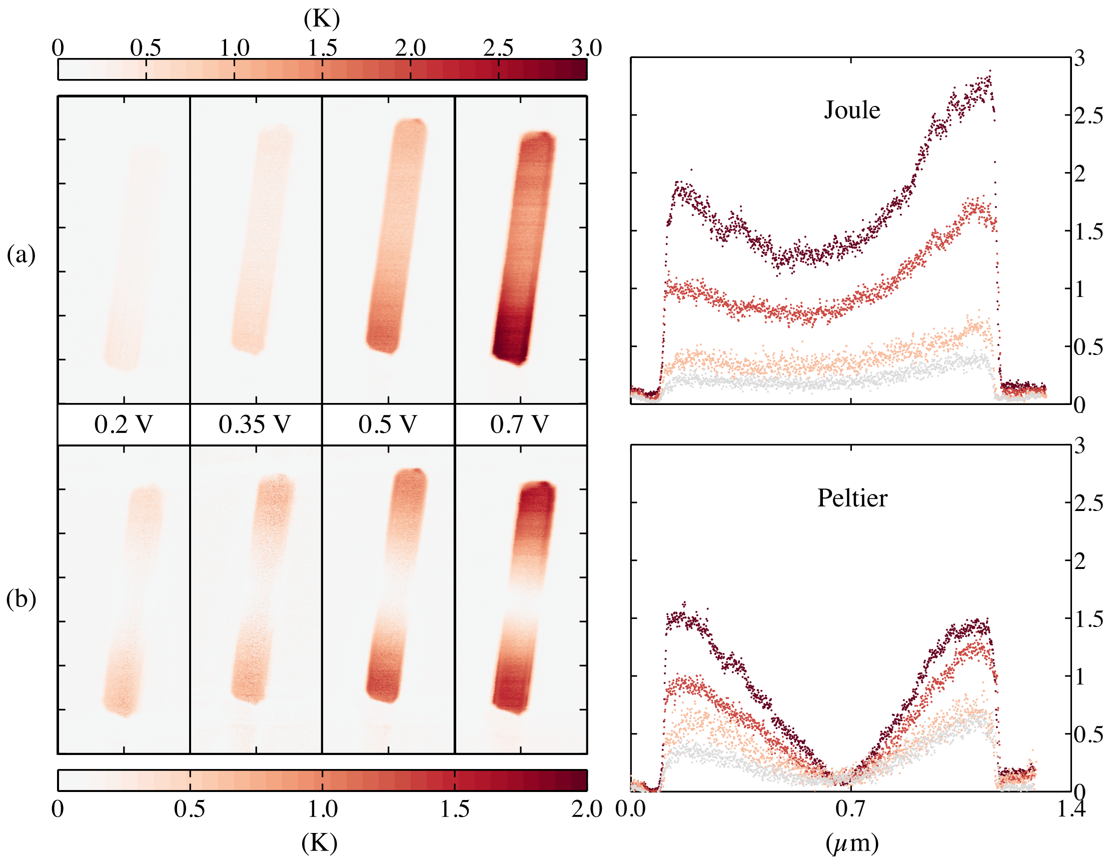


**Supplementary Figure 2. Joule and Peltier temperature as function of the voltage bias**
(a) Steady-state ($\Delta T_{\mathrm{sample,DC}}$) temperature increase at different voltage bias amplitudes and corresponding temperature profiles along the nanowire length direction.
(b) Peltier temperature amplitude ($\Delta T_{\mathrm{sample,1f}}$) at different voltage bias amplitudes and corresponding temperature profiles along the nanowire length direction.

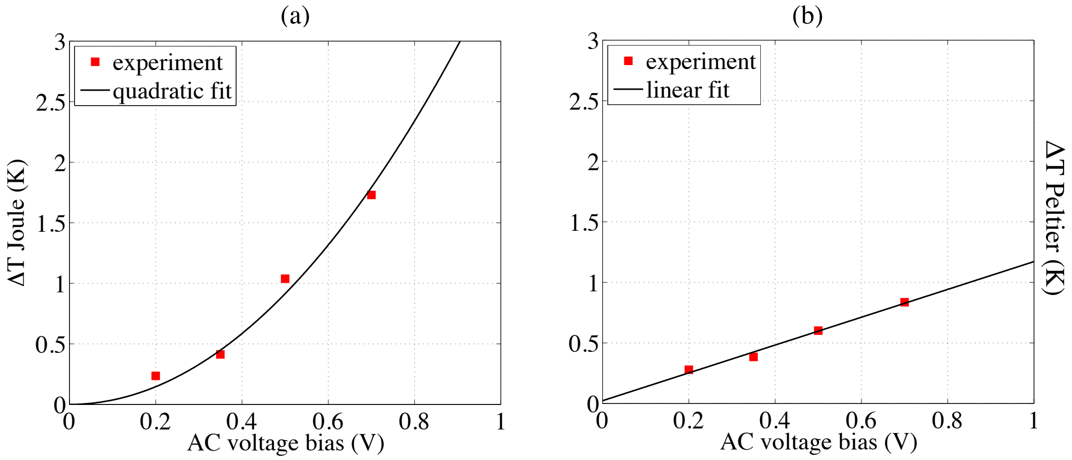


**Supplementary Figure 3. Mean temperature as function of voltage bias**

(a) Average steady-state temperature ($\Delta T_{\mathrm{sample,DC}}$) increase of the nanowire as function of the voltage bias. The fit illustrates a quadratic scaling as is typical for Joule heating.

(b) Average Peltier temperature amplitude ($\Delta T_{\mathrm{sample,1f}}$) of the nanowire as function of the voltage bias. The fit illustrates a linear scaling as is typical for Peltier heating/cooling.

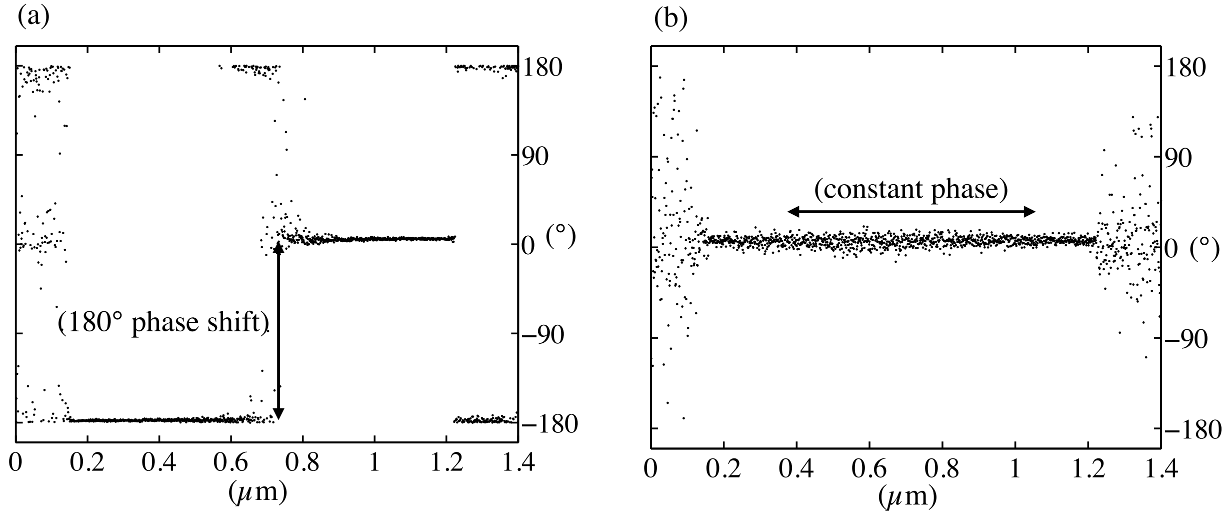


**Supplementary Figure** 4. **Phase signal profiles along the nanowire.**

(a) For ($V_{Lev,1}$), showing a 180° phase shift in the nanowire center that corresponds to the bias-polarity dependence of the Peltier effects on the two opposite metal-InAs contacts.

(b) For ($V_{Lev,2}$), showing a constant phase signal in relation to Joule heating.

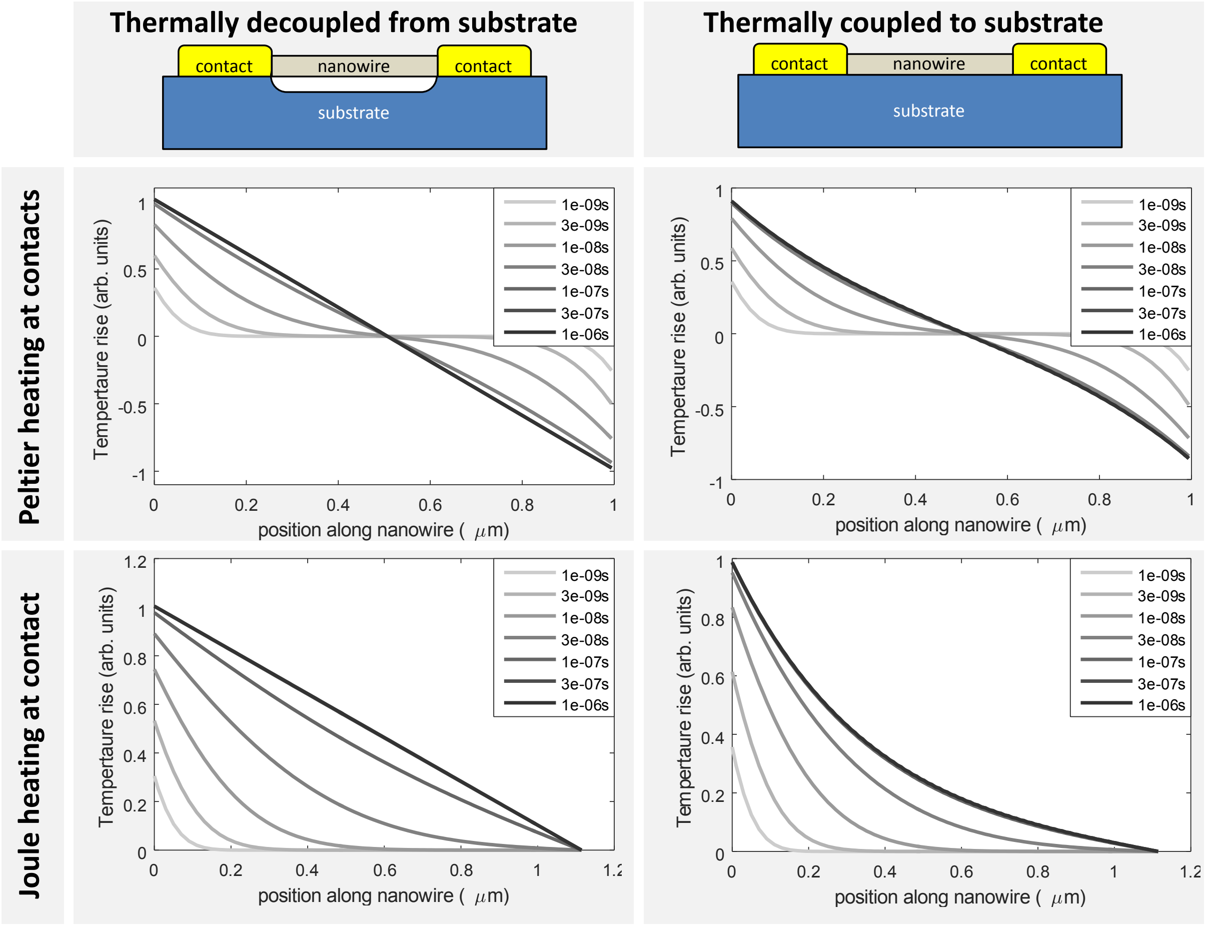


**Supplementary Figure 5. Simulated transient temperature response of the nanowire** Distributions of temperature along an InAs nanowire are simulated as function of time for a nanowire decoupled from the substrate and a nanowire coupled to the substrate. Both, for Peltier heating/cooling at the contacts as well as Joule heating at one of the contacts, thermal equilibrium is established within one microsecond or less.

## SUPPLEMENTARY NOTE 1: RELATION BETWEEN THE SAMPLE TEMPERATURE AND MEASURED ELECTRICAL QUANTITIES

In the following, we derive the relation between the sample temperature and the measured electrical quantities in the scanning probe sensor. Inside a sample, the Joule heat flux scales with the square of the electric current $I$ and the electrical resistance $R$ as

$$\dot{Q}_{\mathrm{Joule}} = R\, I^2 \quad , \tag{1}$$

while the Peltier heat, in contrast, scales linearly with current as

$$\dot{Q}_{\mathrm{Peltier}} = \Pi\, I \quad , \tag{2}$$

where $\Pi$ is the Peltier coefficient . In steady state, accordingly, the temperature distribution in the sample consists of components scaling with $I$ and $I^2$ . During the experiment, a sinusoidal current is applied

$$I = I_0 \sin(\omega t) \, , \tag{3}$$

leading to a modulation of the sample temperature

$$T_{\mathrm{sample}} = T_{\mathrm{sample,DC}} + T_{\mathrm{sample,1f}} \sin(\omega t) + T_{\mathrm{sample,2f}} \sin(2\omega t) \, , \tag{4}$$

with $T_{\mathrm{sample,1f}}$ being proportional to the Peltier effect ($1\omega$) and $T_{\mathrm{sample,2f}}$ being proportional to the Joule temperature modulation ($2\omega$). The steady-state temperature of the sample $T_{\mathrm{sample,DC}}$ is related to the Joule heating of the sample and can be estimated [1] for an applied excitation frequency ($\omega$) as

$$T_{\mathrm{sample,DC}} = \sqrt{1 + (2\omega\tau)^2} \times T_{\mathrm{sample,2f}} + T_{\mathrm{RT}} \tag{5}$$

where $\tau$ is the thermal time constant of the device and $T_{\mathrm{RT}}$ denoting room temperature. The thermal time constant $\tau$ can be derived experimentally from frequency-dependent measurements of the temperature field or estimated using models or simulations. For nanostructures such as those described in this paper, typical time constants are between 10 ns and 10 $\mu$s. If the excitation frequency ($\omega = 2\pi f$) is chosen small enough with respect to $\tau$, so that the device temperature is independent of the excitation frequency [2], we can approximate $T_{\mathrm{sample,DC}} \approx T_{\mathrm{sample,2f}} + T_{\mathrm{RT}}$. This is the case in the experiments described here.

The variations of the sample temperature give rise to changes in the tip-sample heat flux ($\dot{Q}_{\mathrm{ts}}$):

$$\dot{Q}_{\mathrm{ts}} = \dot{Q}_{\mathrm{ts,DC}} + \dot{Q}_{\mathrm{ts,1f}} \sin(\omega t) + \dot{Q}_{\mathrm{ts,2f}} \sin(2\omega t)\,, \tag{6}$$

and to changes of the sensor temperature ($T_{\mathrm{sensor}}$),

$$T_{\mathrm{sensor}} = T_{\mathrm{sensor,DC}} + T_{\mathrm{sensor,1f}} \sin(\omega t) + T_{\mathrm{sensor,2f}} \sin(2\omega t)\,, \tag{7}$$

which are detected in the scanning probe sensor. Note that $T_{\mathrm{sensor,DC}}$ is induced not only by the interaction with the sample via $\dot{Q}_{\mathrm{ts}}$, but mainly results from a steady-state heating of the sensor. For the Joule component, they are related to changes of the sample temperature as

$$T_{\mathrm{sample,DC}} - T_{\mathrm{RT}} = T_{\mathrm{sample,2f}} = \frac{T_{\mathrm{sensor,2f}}\dot{Q}_{\mathrm{ts,DC}} - (T_{\mathrm{sensor,DC}} - T_{\mathrm{RT}})\dot{Q}_{\mathrm{ts,2f}}}{\dot{Q}_{\mathrm{ts,DC}} - \dot{Q}_{\mathrm{ts,2f}}}. \tag{8}$$

For the Peltier component, they are related to changes of the sample temperature as

$$T_{\mathrm{sample,1f}} = \frac{T_{\mathrm{sensor,1f}}\dot{Q}_{\mathrm{ts,DC}} - (T_{\mathrm{sensor,DC}} - T_{\mathrm{RT}})\dot{Q}_{\mathrm{ts,1f}}}{\dot{Q}_{\mathrm{ts,DC}} - \dot{Q}_{\mathrm{ts,1f}}}. \tag{9}$$

From Supplementary Equation(8) and Supplementary Equation(9), it follows that the sample temperature fields in response to Joule heating and the Peltier effect are determined by the AC ($1f$, $2f$) and DC components of the sensor-sample heat flux ($\dot{Q}_{\mathrm{ts}}$) and the sensor temperature ($T_{\mathrm{sensor}}$).

How these quantities are extracted from the measurement data depends on the operation mode of the SThM. Often, constant probing current or constant sensor temperature modes are used. In our experiments, however, we apply a constant voltage to the heater/sensor in series with a series resistor and measure the voltage response of the heater/sensor ($V_{\mathrm{Lev}}$). Accordingly, the electrical voltage bias measured across the scanning probe sensor has various components and can be described as

$$V_{\mathrm{Lev}} = V_{\mathrm{Lev,DC}} + V_{\mathrm{Lev,1f}} \sin(\omega t) + V_{\mathrm{Lev,2f}} \sin(2\omega t)\,. \tag{10}$$

These three different components of the voltage bias can be measured using a differential amplifier to sensitively measure the DC component ($V_{\mathrm{Lev,DC}}$) and two lock-in amplifiers to sense the two AC components in the first ($V_{\mathrm{Lev,1f}}$) and second harmonic response ($V_{\mathrm{Lev,2f}}$), respectively (see Supplementary Figure. 1). To relate these voltage signals to the temperature of the sensor ($T_{\mathrm{sensor}}$) requires calibration of the scanning probe first to establish the

relation between the temperature and the electrical resistance $R$ of the sensor. Calibration of the thermoresisitve probe has already been discussed [3, 4], with an uncertainty in relating the electrical resistance of the probe sensor to temperature of 20%. The uncertainty is mainly caused by simplifying assumptions on the thermal properties of the cantilever as discussed in detail previously [3, 4]. This relative uncertainty feeds linearly into the derived sample temperature without compromising the demonstrated high sensitivity and the spatial distribution of temperature.

We can derive the sensor temperature as a function of $V_{\mathrm{Lev}}$ as

$$T_{\mathrm{sensor,1f}} \approx \left.\frac{\partial T_{\mathrm{sensor}}}{\partial R}\right|_{\mathrm{R0}} \times \left.\frac{\partial R}{\partial V_{\mathrm{Lev}}}\right|_{V_{\mathrm{Lev,DC}}} \times V_{\mathrm{Lev,1f}} \quad \text{and} \tag{11}$$

$$T_{\mathrm{sensor,2f}} \approx \left.\frac{\partial T_{\mathrm{sensor}}}{\partial R}\right|_{\mathrm{R0}} \times \left.\frac{\partial R}{\partial V_{\mathrm{Lev}}}\right|_{V_{\mathrm{Lev,DC}}} \times V_{\mathrm{Lev,2f}} \ . \tag{12}$$

Here, $R_0$ is the resistance measured for $T_{\mathrm{sensor,DC}}$ at $V_{\mathrm{Lev,DC}}$. The approximation used in Supplementary Eqs.(11) and (12) is a linearization $R(T)$ around $R_0$. This is justified because $V_{\mathrm{Lev,1f}}$ and $V_{\mathrm{Lev,2f}}$ are sufficiently small in our experiments. This is the case in our measurements, as the thermal resistance of the cantilever ($R_{\mathrm{cl}} \sim 2\times10^5$ K/W), is typically three orders of magnitudes smaller than the thermal resistance of the tip-sample contact ($R_{\mathrm{ts}} \sim 10^8$ K/W) [5].

Next, the heat flux $\dot{Q}_{\mathrm{ts}}$ between the scanning probe sensor and the sample needs to be related to the electrical measurement signals as well. For this, we need to consider the heat dissipated by the current applied to the heater/sensor via application of a voltage over the heater/sensor and a series resistor $R_{\mathrm{series}}$. The electrical power ($P_{\mathrm{el}}$) dissipated in the cantilever can be directly expressed as a function of the cantilever voltage ($V_{\mathrm{Lev}}$):

$$P_{\mathrm{el}} = \frac{1}{R_{\mathrm{series}}} \left[V_{\mathrm{Tot}} V_{\mathrm{Lev}} - V_{\mathrm{Lev^2}}\right], \tag{13}$$

with $V_{\mathrm{Tot}}$ being the voltage bias applied to the Wheatstone bridge and $R_{\mathrm{series}}$ being the 2 kΩ series resistor in the Wheatstone bridge measurement circuitry of our experiment (see Supplementary Figure. 1). We assume that all electrical power dissipated in the cantilever is dissipated as heat in the cantilever sensor. For steady-state, energy conservation in the heater/sensor requires that the total heat flux into and out of the sensor be equated:

$$P_{\mathrm{el}} = \dot{Q}_{\mathrm{ts}} + \dot{Q}_{\mathrm{Lev}}, \tag{14}$$

where $\dot{Q}_{\text{Lev}}$ is the heat flux through the cantilever towards the room-temperature reservoir and $\dot{Q}_{\text{ts}}$ is the heat flux into the sample at temperature $T_{\text{sample}}$ through the tip-sample contact.

By inserting Supplementary Equation(10) into Supplementary Equation(13) and separating $P_{\text{el}}$ into its various temporal components

$$
\begin{aligned}
P_{\text{el}} = P_{\text{el,DC}} + P_{\text{el,1f}} \sin(wt) + P_{\text{el,2f}} \sin(2\omega t) + P_{\text{el,3f}} \sin(3\omega t) + P_{\text{el,4f}} \sin(4\omega t) \qquad (15)\\
P_{\text{el,DC}} = \frac{1}{R_{\text{series}}} \left[ V_{\text{Tot}} V_{\text{Lev,DC}} - V_{\text{Lev,DC}^2} - \frac{1}{2} V_{\text{Lev,1f}^2} - \frac{1}{2} V_{\text{Lev,2f}^2} \right]\\
P_{\text{el,1f}} = \frac{1}{R_{\text{series}}} \left[ V_{\text{Tot}} V_{\text{Lev,1f}} - 2 \times V_{\text{Lev,DC}} V_{\text{Lev,1f}} - V_{\text{Lev,1f}} V \text{Lev, 2f} \right]\\
P_{\text{el,2f}} = \frac{1}{R_{\text{series}}} \left[ V_{\text{Tot}} V_{\text{Lev,2f}} - 2 \times V_{\text{Lev,DC}} V_{\text{Lev,2f}} - \frac{1}{2} V_{\text{Lev,1f}^2} \right]\\
P_{\text{el,3f}} = \frac{1}{R_{\text{series}}} \left[ V_{\text{Lev,1f}} V_{\text{Lev,2f}} \right]\\
P_{\text{el,4f}} = \frac{1}{R_{\text{series}}} \left[ \frac{1}{2} V_{\text{Lev,2f}^2} \right]
\end{aligned}
$$

we can derive the electrical power dissipated in the heater/sensor of the scanning probe. Note that the higher-order terms at $(3\,f)$ and $(4\,f)$ are very small and therefore are not considered for further analysis. To derive $\dot{Q}_{\text{ts}}$ using Supplementary Equation(14), we need the heat flux into only the cantilever ($\dot{Q}_{\text{Lev}}$), which can be calculated using the sensor temperature ($T_{\text{sensor}}(V_{\text{Lev}})$ of Supplementary Equation(7):

$$
\begin{aligned}
\dot{Q}_{\text{Lev}} = \dot{Q}_{\text{Lev,DC}} + \dot{Q}_{\text{Lev,1f}} \sin(wt) + \dot{Q}_{\text{Lev,2f}} \sin(2wt), \quad \text{with} \qquad (16)\\
\dot{Q}_{\text{Lev,DC}} = \frac{T_{\text{sensor,DC}} - T_{\text{RT}}}{R_{\text{Lev}}}\\
\dot{Q}_{\text{Lev,1f}} = \frac{T_{\text{sensor,1f}}}{R_{\text{Lev}}}\\
\dot{Q}_{\text{Lev,2f}} = \frac{T_{\text{sensor,2f}}}{R_{\text{Lev}}}.
\end{aligned}
$$

Here, only the thermal resistance of only the cantilever ($R_{\text{Lev}}$) is needed and has to be derived prior the measurement scan from tip-sample approach curves as described elsewhere [4]. By inserting Supplementary Equation(15) and Supplementary Equation(16) into Supplementary Equation(14), and Supplementary Equation(14) and Supplementary Equation(11),(12) into Supplementary Equation(8) and Supplementary Equation(9), respectively, we can finally express, $T_{\text{sample}}$ as function of the DC and AC voltage components of $V_{\text{Lev}}$ measured across the cantilever.

The assignment of the temperature signals at $1f$ and $2f$ to heating/cooling due to the Peltier effect and Joule heating, respectively, inherently assumes that the electrical resistance of the device is sufficiently independent of the bias or current applied, which is the case in our experiments. If, in contrast, a diode behaviour were observed in the device, then the Joule dissipation would also contribute to $T_{\mathrm{sample,1f}}$. However, using the $I - V$ characteristics of the device, the relation of Joule and Peltier effects may still be recovered through a variation of the amplitude and voltage offset.

## SUPPLEMENTARY NOTE 2: BIAS-DEPENDENCE OF OF JOULE AND PELTIER DISTRIBUTIONS OF TEMPERATURE

We investigate the voltage bias dependence of the temperature of the nanowire. For a reasonably linear device resistance, the device current (3) is proportional to the applied voltage and the Joule (Supplementary Equation(1)) and Peltier (Supplementary Equation(2)) heat depend quadraticaly and linearly on the applied voltage, respectively.

To demonstrate this scaling dependence, four different AC voltage bias excitation amplitudes were applied to the nanowire, and the temperature field components were characterized as discussed in the main paper. Supplementary Figure. 2(a) shows the AC steady-state temperature increase ($\Delta T_{\mathrm{sample,2f}}$) due to Joule heating and Supplementary Figure. 2(b) the simultaneously recorded Peltier temperature amplitude ($\Delta T_{\mathrm{sample,1f}}$). Note that the Peltier temperature field ($\Delta T_{\mathrm{sample,1f}}$) is now plotted as amplitude signal only because the phase information is not needed to illustrate the bias-dependent evolution of the Peltier temperature in comparison with the steady-state temperature increase ($\Delta T_{\mathrm{sample,DC}}$). For a low excitation bias of 0.2 V, we observe a very small temperature increase attributed to Joule heating, which is only just significant. In contrast, at this voltage, the Peltier heating/cooling is considerably larger. With increasing voltage bias, the trend reverses and the Joule heating exceeds Peltier cooling/heating at the contacts.

This can easily be seen from the thermal images and the temperature profiles extracted along the nanowire, with each data point being an average of a 20-nm-wide section on the flat top of the nanowire. The maximum Joule temperature on the right contact increases from about 0.4 K to 2.8 K, while the Peltier temperature amplitude simultaneously increases from 0.6 K to 1.4 K. The Peltier temperature amplitude exceeds the steady-state temperature

increase ($\Delta T_{\mathrm{sample,DC}}$) at low voltage bias. Accordingly, parts of the nanowire can temporally cool below ambient temperature within one excitation period as the Joule temperature modulation around the steady-state temperature gets zero at the maximum amplitude of the Peltier temperature modulation. The appearance of temporal cooling effects at the nanowire contacts in a voltage bias regime between about ∼0 and $0.6\,\mathrm{V_{pp}}$ is an interesting finding, as this is the supply voltage bias range forseen for future nanowire electronic devices [6]. Peltier effects are likely to dominate over Joule heating in low-power nanoelectronics, highlighting the importance of investigating thermal interface effects experimentally.

To illustrate this clearly, we plotted the average Joule temperature increase along the nanowire and the average Peltier temperature amplitude as a function of the applied voltage bias in Supplementary Figure. 3. Each experimental data point corresponds to an average temperature amplitude calculated as the mean of the temperature amplitude profiles along the nanowire segment in Supplementary Figure. 2. The fit to the experimental data points in Supplementary Figure. 3(a) shows a quadratic dependence of the second harmonic temperature increase, whereas the fit in Supplementary Figure. 3(b) illustrates a linear dependency of the measured first harmonic temperature signal as function of the applied voltage bias. These two scaling dependencies support our conclusion that pure Joule heating is observed in the second harmonic and Peltier heating/cooling in the first harmonic response of the scanning probe sensor.

## SUPPLEMENTARY NOTE 3: PHASE INFORMATION FROM LOCK-IN MEASUREMENTS

The temporal heating and cooling due to the Peltier effects at the metal/InAs contacts are related to a phase shift in the raw voltage signal acquired by the scanning probe sensor. Supplementary Figure. 4 shows the phase signal along the nanowire length-direction as observed in the first harmonic response ($V_{\mathrm{Lev},1}$) of the scanning probe sensor.

The bias-polarity dependence of the Peltier effects at the opposite metal/InAs contacts corresponds to a $180\,^{\circ}$ phase shift at the nanowire center at vanishing signal amplitude (see Fig.3 (c) in the main article). In contrast, we observe a constant phase signal along the nanowire length direction in the second harmonic ($V_{\mathrm{Lev},2}$) that is related to Joule heating of

the nanowire.

## SUPPLEMENTARY DISCUSSION: TRANSIENT TEMPERATURE RESPONSE OF THE NANOWIRE

Our experimental data in the main manuscript is interpreted assuming that the instantaneous temperature rise due to an applied AC voltage bias is well approximated by the steady state reached by applying a corresponding DC current (or voltage) to the device. Here, we discuss the limits of this assumption on one hand, and possibilities of observing transient thermal effects, on the other hand.

For observation of steady-state behaviour, the experimental time scale, given by $\omega^{-1}$, needs to be much larger than the internal equilibration time of the active device, $\tau$ (see Supplementary Equation 5). If the criterion $\omega^{-1} \gg \tau$ holds, then the thermal measurement signals should be independent of a variation of $\omega^{-1}$, and the time-averaged DC temperature rise above room-temperature ($T_{RT}$) equals the maximum amplitude of temperature modulation ($T_{\text{sample,DC}} \approx T_{\text{sample,2f}} + T_{\text{RT}}$). Experimentally, the intrinsic time scale of an electronic device can oftentimes be determined directly from the time response of an applied voltage or current. In our experiments, we observed no significant signal dependence on the choice of $\omega$ in the range up to the roll-off frequency of our probe sensor (100 kHz). We note that the intrinsic thermal time constant of our scanning probe sensor is typically the limiting time constant in our experiments (see main manuscript).

Alternatively to the direct experimental determination, the thermal time constant of the device can also be determined from simulation provided all relevant parameters are known. Here, we provide a simulation to discuss, on the one hand, the case when criterion $\omega^{-1} \gg \tau$ does not hold and transient device behaviour might be observed, and on the other hand to support our conclusion of working in the regime of $\omega^{-1} \gg \tau$ in our experiments.

To simulate the transient device behaviour, we solve the heat equation (sometimes also called heat diffusion equation) numerically for our nanowire system. From what is known about the InAs nanowires employed in this study, the average room-temperature phonon mean free path (the dominant heat carriers in semiconductors) is much smaller than the length of the nanowire ($\approx$ $1.2\,\mu m$) [13]. Therefore, a diffusive transport model can to be

applied [7, 8]. Furthermore, given the aspect ratio of the nanowire and the fact that the mean free path may be up to a magnitude similar to the diameter of the nanowire, it is appropriate to approximate the system as a one-dimensional system [7, 9–11]. The heat equation was solved on finite differences using a standard implementation in Matlab and applying the routine tests for numerical accuracy. The materials parameters for the system are known within some uncertainty. The largest unknown is the coupling of the nanowire to the substrate. Therefore we decided to model two cases, a completely thermally decoupled nanowire as one limiting case, and a nanowire coupled to the substrate with a coupling strength (thermal conductance of $0.3\,\mathrm{W(Km)^{-1}}$ per unit length) taken from a previous experimental example [4]. The other parameters chosen are a thermal conductivity of the nanowire of $4\,\mathrm{W(Km)^{-1}}$ (reported values between 2-10 $\mathrm{W(mK)^{-1}}$ [12, 13]), and a diffusivity of $2.8 \times 10{-}6\,\mathrm{m}^2 s^{-1}$. Using other values within this thermal conductivity span did not change the conclusion presented below. The thermal contact to the electrode was modeled with an appropriate thermal contact as discussed elsewhere [9, 11].

Simulation results for four different cases are shown in Supplementary Figure. 5. One case is a symmetric heating/cooling as expected from the Peltier effects at the two contacts, another case is a heating at one of the contacts simulating a 'bad' electrical contact as attributed to the results from our experiment (see main manuscript). (A third case illustrating the typical parabolic Joule heating along the nanowire is well documented in literature and not reproduced here.) The lines plotted are temperature distributions along the nanowire for different times. At short time scales, the approximately exponential temperature profiles are observed as expected from text book calculations [14, 15]. After some time, however, the heat wave extends over the entire nanowire and converges towards a certain steady state.

In all cases the steady state is reached within less than $1\,\mu$s and in most cases faster than 100 ns. Likewise, the thermal time constant associated with the heat spreading into the carrier substrate is calculated to be faster than $1\,\mu$s (not shown). The simulation results of the transient behaviour can be compared to the experimental data to discuss whether the observed distribution of temperature can be expected within the interpretation of a steady-state behaviour. For example, a well decoupled nanowire with good contacts (both electrically and thermally) would lead to a linear steady state temperature distribution due to Peltier heating and a parabolic distribution due to Joule heating within the wire.

With increasing thermal coupling between nanowire and substrate, the linear temperature

profile due to Peltier heating at the contacts should gradually change towards a decay function from the contacts into the nanowire. However, the same observation would be made if the time constant of the experiment was comparable or faster than the intrinsic device thermal time constant. This demonstrates (using the case of a clamped nanowire) that our thermometry method requires some basic knowledge (gained from experiments or simulations) on the thermal time constants for an accurate interpretation of the spatially resolved thermometry data.

With regard to our data, let's consider the case of Joule heating along the nanowire. Joule heating within the nanowire leads to a parabolic temperature distribution when the wire is well decoupled from the substrate and well coupled to the electrodes. With increasing coupling to the substrate, the temperature distribution flattens until it reaches a constant value except very near the contacts [10]. Therefore, regardless of substrate coupling, there should be a maximum in the temperature distribution in the center of the wire in steady state. The temperature distribution attributed to Joule heating in our experiments, however, exhibits a minimum in the nanowire center, in contrast to the parabolic temperature distribution observed along the self-heated metal interconnect, which hints to dominant heating from the electrical contacts, similar to the simulations in Supplementary Figure. 5. For this particular nanowire sample, we conclude that heating from the contact regions dominates over Joule heating along the nanowire, and that the observation is not an artifact caused by the chosen modulation frequency.

Finally, we would like to stress, that from various nanoscale samples we have been testing in our lab, we may conclude that the time constants chosen in our experiments can be safely applied in almost all situations and are well compatible with the typical operation speed of scanning probe microscopes. If the steady state temperature field of microscopic or even well decoupled MEMS structures with time constants on the order of 1 kHz or less should be studied, scanning speed needs to be reduced accordingly. On the other hand, applying our proposed thermometry methods in a regime where steady state is not reached, will allow conclusions on transient device behaviour, a better localization of heat sources, and extracting diffusivities from measured data. This will be done in future studies.

## SUPPLEMENTARY REFERENCES


[1] Banerjee, K. *et al.* Investigation of self-heating phenomenon in small geometry vias using scanning joule expansion microscopy. *Reliability Physics Symposium Proceedings* **37**, 297–302 (1999).

[2] Bontempi, A., Thiery, L. & Teyssieux, D. DC and AC scanning thermal microscopy using micro-thermoelectric probe. *High Temperatures-High Pressures* **46**, 321–332 (2014).

[3] Nelson, B. Temperature calibration of heated silicon atomic force microscope cantilevers. *Sensors and Actuators A: Physical***140**, 51–601 (2007)59

[4] Menges, F., Riel, H., Stemmer, A. & Gotsmann, B. Quantitative Thermometry of Nanoscale Hot Spots. *Nano Letters* **12**, 596–601 (2012).

[5] Menges, F., Riel, H., Stemmer, D. C., A & Gotsmann, B. Thermal Transport into Graphene through Nanoscopic Contacts. *Physical Review Letters* **111**, 205901 (2013).

[6] Ionescu, A. M. & Riel, H. Tunnel field-effect transistors as energy-efficient electronic switches. *Nature* **479**, 329–337 (2011).

[7] Volz, S. *Microscale and nanoscale heat transfer*, vol. 1 (Microscale and Nanoscale Heat Transfer, 2007).

[8] Chen, G. *Nanoscale energy transport and conversion* (Oxford University Press, 2015).

[9] Mensch, P. F.-J. *Thermoelectric Characterization of InAs Nanowires.* ETH Zurich (2015). URL `http://dx.doi.org/10.3929/ethz-a-010526730`.

[10] Völklein, F., Reith, H., Cornelius, T. W., Rauber, M. & Neumann, R. The experimental investigation of thermal conductivity and the Wiedemann–Franz law for single metallic nanowires. *Nanotechnology* **20**, 325706 (2009).

[11] Karg, S. *et al.* Measurement of Thermoelectric Properties of Single Semiconductor Nanowires. *Journal of Electronic Materials* **42**, 2409–2414 (2013).

[12] Karg, S. F. *et al.* Full thermoelectric characterization of InAs nanowires using MEMS heater/sensors. *Nanotechnology* **83**, 305702 (2014).

[13] Zhou, F., Moore, A. L., Bolinsson, J., Persson, A. & Fröberg, L. Thermal conductivity of indium arsenide nanowires with wurtzite and zinc blende phases. *Physical Review B* **25**,

205416 (2011).

[14] Lienhard, J. H. & Lienhard, J. H. *A heat Transfer Textbook, 14th ed.,* (Phlogiston Press, Cambridge MA, 2011).

[15] Winiewski, S. & Nowak, W. Transient heat conduction in semi-infinite solid with specified surface temperature. In Hetnarski, R. (ed.) *Encyclopedia of Thermal Stresses*, 6171–6180 (Springer Netherlands, 2014).
